# Phase I study of the safety, tolerability, and potential therapeutic dose of OMT-110 for patients with refractory metastatic Colorectal Cancer

**DOI:** 10.1186/s12885-025-14351-1

**Published:** 2025-05-25

**Authors:** Youngbae Jeon, MinJeong Jung, BongHwang Jeong, Haejun Lee, Sun Jin Sym, Jeong-Heum Baek

**Affiliations:** 1 Division of Colorectal Surgery, Department of Surgery, Gachon University Gil Medical Center, Incheon, Republic of Korea; 2 College of Pharmacy, Gachon University, Incheon, Republic of Korea; 3 Department of Statistics, LSK Global Pharma Services Co., Ltd, Seoul, Republic of Korea; 4 Department of Nuclear Medicine, Gachon University Gil Medical Center, Incheon, Republic of Korea; 5 Division of Hematology and Oncology, Department of Internal Medicine, Gachon University Gil Medical Center, Incheon, Republic of Korea; 6 Gachon University College of Medicine, Incheon, Republic of Korea; 7 Division of Colon and Rectal Surgery, Department of Surgery, Gil Medical Center, Gachon University College of Medicine, 21, Namdong-daero 774 Beon-gil, Namdong-gu, Incheon, 21565 Republic of Korea

**Keywords:** OMT-110, Colorectal neoplasm, Neoplasm metastasis, Phase I, Safety, Chemotherapy

## Abstract

**Background:**

OMT-110 is a repositioned drug candidate for the treatment of metastatic colorectal cancer (mCRC). This phase I study aimed to determine the appropriate dose of OMT-110 for phase II trials and its safety, tolerability, and efficacy. We conducted the first-in-human dose-escalation study of patients with advanced mCRC (age 20 years or older) who had refractory disease.

**Methods:**

OMT-110 was administered subcutaneously in a repeated cycle of 21 days on/seven days off until tumor progression, toxicity, or withdrawal. Adverse events were graded according to the National Cancer Institute Common Terminology Criteria ver4.03. The pharmacokinetic profiles were determined before and after OMT-110 administration. Pharmacodynamic and efficacy evaluations were performed using abdominopelvic computed tomography (APCT), chest CT, and ^18^F-FDG-Positron emission tomography/CT, following the Response Evaluation Criteria in Solid Tumors v1.1. Fourteen patients were divided into four cohorts to receive doses ranging from 12.5 mg to 100 mg daily.

**Results:**

Based on our results, a daily dose of 100 mg is recommended following a repeated 28-day treatment cycle of 21 days on/seven days off. Of the 54 adverse events experienced by the participants in the safety set, all were grade 1 or 2, except for two serious adverse events. OMT-110 was either “unrelated” or “definitely not related” or “probably unrelated” to these events. Pharmacokinetic analysis revealed no apparent accumulation.

**Conclusions:**

Based on the evaluation of pharmacodynamic and efficacy parameters, OMT-110 is a promising novel systemic therapy with potential immunomodulatory effects for patients with advanced mCRC.

**Trial registration:**

CRIS Registration Number KCT0005336 (first posted on 08/08/2017).

## Introduction

Colorectal cancer is the third most common cancer worldwide, accounting for approximately 10% of all malignancies, and the second leading cause of cancer-related deaths globally [1]. Patients with metastatic colorectal cancer (mCRC) typically receive first- and second-line treatments, including cytotoxic agents (5-fluorouracil, oxaliplatin, and irinotecan), biologically targeted agents (cetuximab and bevacizumab), and immune checkpoint inhibitors for mismatch repair deficient/microsatellite instability-high [2]. Although disease control and enhanced life expectancy remain the therapeutic goals when patients receive the third line of treatment or beyond [3], standard therapies for mCRC have not been firmly established. Although regorafenib and TAS-102 have been approved for third-line treatment, their limited efficacy and safety profiles (e.g., myelotoxicity, hand-foot syndrome) highlight the need for new therapeutic strategies. [4, 5].

The tumor microenvironment (TME) of solid tumors plays a vital role in tumor progression and immunosuppression [6–8]. The TME of solid tumors is characterized by several features, such as hypoxia, inflammation, and fibrosis. OMT-110 is a small-molecule candidate that can be repositioned for the treatment of solid tumors. Initially, OMT-110 was considered a candidate for improving the hypoxic microenvironment of solid tumors and inducing a shift in the energetic metabolism of cancer cells from aerobic glycolysis to mitochondrial aerobic metabolism by downregulating hypoxia-inducible factor-1α [9, 10]. According to preclinical studies, OMT-110 is a potent inhibitor of several angiogenic and stromal RTKs, including vascular endothelial growth factor (VEGFR)-1, VEGFR-2, VEGFR-3, platelet-derived growth factor receptor (PDGFR)-b, fibroblast growth factor receptor 1, and TIE2 [11, 12]. Among the various effects of OMT-110, its efficacy in human colorectal cancer with epidermal growth factor receptor (EGFR) mutations and sufficient safety margins in terms of dosage have been previously confirmed [11, 13]. However, recent findings have shown that OMT-110 causes immunogenic cell death, increases the immunogenicity of cancer cells, and further improves the immunosuppressive TME, including the fibrotic defense against immune cells [11, 14, 15, 16]. Accordingly, OMT-110 could be used be used as a comprehensive monotherapy or as part of a combination regimen with conventional treatments such as chemotherapy, radiotherapy, and emerging immunotherapies for solid tumors.

Here, we report the first-in-human phase I single-agent dose escalation study in patients with mCRC who were unresponsive to conventional treatments. This study aimed to define the safety profile and tolerability of OMT-110 in patients with refractory mCRC and to determine the recommended dose for a phase II study. Pharmacokinetic-pharmacodynamic parameters and tumor responses were evaluated as secondary objectives to assess the efficacy of OMT-110.

## Materials and methods

### Study design and performance

This study was designed as prospective, open-label, single-arm, and dose-escalating protocol. OMT-110 was added to a 20 mg/mL vial. The following doses of OMT-110 were administered: 12.5 mg/day (cohort 1), 25 mg/day (cohort 2), 50 mg/day (cohort 3), and 100 mg/day (cohort 4). A standard 3 + 3 design was used for the dose escalation [17]. If dose-limiting toxicity (DLT) occurred in two or more of the three patients at the initially administered dose (12.5 mg/0.625 ml), the clinical trial was terminated. If a DLT occurred in one of the three patients, the DLT was evaluated in an expanded cohort of six patients, with the inclusion of three additional patients. If a DLT occurred in one of the six patients, the dose was escalated to the next level. If there were two or more DLTs in three patients, the evaluation of DLT was conducted in an expanded cohort of six patients, with the inclusion of three additional patients at a lower dose level. If six patients had already been administered level one below, additional patients were not enrolled, and the dose at this level was defined as the maximum tolerated dose (MTD).

Each cohort was comprised of four cycles. Each cycle consisted of three consecutive weeks of daily subcutaneous injections, followed by a treatment-free period of one week. The principal investigator (PI) delayed the administration frequency, but not by more than two weeks. The visit schedule was as follows: pre-dose screening visit, four cycles of treatment, end-of-treatment visit, and follow-up observation (Table 1).

**Table 1:** Dose cohort, treatment duration, and relative dose intensity

Dose cohort	1		2		3		4		Total
Dose (mg/day)	12.5		25		50		100		
Patients, n	4		3		4		3		14
Treatment duration, days^*^									
Median (range)	73 (10–106)		47 (46–48)		35 (13–103)		105 (47–109)		47 (10–109)
Relative dose intensity, %									
Median (range)	96.9 (95.3–100.0)		104.6 (104.6–109.5)		100.0 (84.0–102.2)		99.1 (95.5–104.6)		100.0 (84.0–109.5)

^*^Treatment duration = date of the last day of treatment with the study drug–date of the first day of treatment with the study drug + 1

This study was approved by the Institutional Review Board of the Gachon University Gil Medical Center (authorization number: GCIRB2017-246) and the Ministry of Food and Drug Safety. This study was conducted following the principles of the Declaration of Helsinki. The clinical trial was monitored by an independent Contract Research Organization of LSK Global Pharma Services Co., Ltd. All patients signed a written informed consent form after being advised of the risks, benefits, and alternatives to the study treatments.

### Patient population

The key inclusion criteria were (1) age ≥ 20 years; (2) unresectable metastatic colorectal adenocarcinoma; (3) increase in lesion size despite at least a second line of conventional chemotherapy; (4) Eastern Cooperative Oncology Group (ECOG) performance status 0–2; (5) adequate function of major organs, including hepatic transaminases < 5 times the upper normal limit, bilirubin < 2 times the upper normal limit, serum creatinine < 1.5 × the upper normal limit, platelet count > 100,000/mm^3^, and neutrophil count > 1,500/mm^3^. The key exclusion criteria were (1) history of malignancy except colorectal cancer within the last five years; (2) primary cancer-related complications requiring emergency surgery; (3) American Society of Anesthesiologists physical status ≥ 4; (4) active infection requiring intravenous antibiotic therapy; (5) active cardiovascular disease, including cerebrovascular accident or myocardial infarction within the last six months, unstable angina pectoris, congestive heart failure per New York Heart Association classification ≥ Grade II, and cardiac arrhythmia per electrocardiogram; (6) pregnancy; (7) nephropathy (estimate glomerular filtration rate below 50 mL/min); (8) any other investigative product within the 30 days before the first drug administration; and (9) type 2 uncontrolled diabetes despite standard treatment.

Clinical evaluation, including medical history, physical examination, previous anticancer therapy, ECOG performance status, baseline characteristics, physical measurement, pregnancy test, laboratory test, chest X-ray, electrocardiogram, abdominopelvic computed tomography (APCT), chest CT, ^18^F-FDG positron emission tomography (PET)/CT, and ultrasonography of the kidney/heart, was performed during screening.

### Safety analysis

Planned safety assessments included treatment-emergent adverse event (TEAE) monitoring and evaluation of DLTs and MTD. TEAEs were documented in terms of severity, onset, resolution date, relevance to OMT-110, actions taken for associated adverse events, and outcomes. The severity of TEAEs was graded according to the National Cancer Institute Common Terminology Criteria for Adverse Events (NCI-CTCAE ver4.03). A safety assessment was conducted on patients who received the investigated product (IP) at least once. The TEAEs were standardized using MedDRA and categorized into system organ class and preferred term. The severity of AEs, incidence, and percentage of adverse reactions, drug-related adverse events, and serious adverse events (SAEs) are presented according to the cohort.

DLTs were assessed according to the NCI-CTCAE ver4.03 during the first cycle of each cohort. DLTs were defined as the occurrence of any of the following: absolute neutrophil count (ANC) < 500/mm^3^ and fever $\:\ge\:$38.3 ℃; ANC <500/mm^3^ for $\:\ge\:$8 days; platelet <25,000/mm^3^; thrombocytopenic bleeding, including platelet <50,000 mm^3^; grade $\:\ge\:$3 toxicity (nausea, vomiting, diarrhea, and fatigue), including persistence for $\:\ge\:$3 days despite the use of appropriate remedial measures and the persistence of grade 3 fatigue for $\:\ge\:$8 days; incapable of completing a cycle within six weeks; and incapable of receiving treatment for $\:\ge\:$14 consecutive days. Participants who received less than 75% of the planned number of administrations were excluded from the DLT evaluation population. MTD was defined as the highest dose that could be administered to six patients to ensure that no more than one patient experienced DLT. Interruptions in dosing and dose reduction were permitted if any criteria corresponding to treatment discontinuation were met.

### Pharmacokinetics

The pharmacokinetic assessment included a comparison of the concentrations of calcium and lactate ions released after OMT-110 administration, both pre- and post-administration. Blood samples were collected as follows: cohort 4, 30 min, 1 h, 2 h, 4 h, and 8 h pre-dose on days 1–2 of cycle 1, and 30 min, 1 h, 2 h, 4 h, and 8 h post-dose on days 19–20 of cycle 1. Pharmacokinetic evaluation included the assessment of serum calcium and lactate levels that exceeded baseline concentrations. The evaluation variables included T_max_, C_max_, and AUC_(0−24)_. A pharmacokinetic assessment was conducted on patients in cohort 4 who had received IP at least once and were eligible for pharmacokinetic analysis. Pharmacokinetic parameters were calculated using standard compartmental and non-compartmental analyses. The linearity of the pharmacokinetic parameters with respect to the dose was analyzed using linear regression analysis and the Kruskal–Wallis test for dose-adjusted pharmacokinetic parameters.

### Pharmacodynamics

Considering the mechanism of action of a drug, noninvasive methods, such as FDG-PET/CT, can be used to assess pharmacodynamic parameters without directly collecting tumor samples. FDG-PET was performed as follows: screening, post-dose of cycles 2 and 4, and at the final study visit. Legions of interest for FDG-PET were selected independently from the CT scans. These lesions were analyzed using standard uptake values (SUV) of the signals. SUVs of up to five lesions were evaluated and classified as having a complete metabolic response, a partial metabolic response (PMR), a stable metabolic disease (SMD), or a progressive metabolic disease following the European Organization for Research and Treatment of Cancer.

### Efficacy

Efficacy was evaluated in patients who received at least one dose of OMT-110 and were eligible for tumor response assessment (i.e., patients with at least one follow-up evaluation). The following efficacy analysis populations were developed: the Per-Protocol Set (PPS), consisting of patients who underwent tumor response evaluation after completing cycle 2, and the Full Analysis Set (FAS), consisting of patients who underwent tumor response evaluation. Tumor response was assessed using chest CT and APCT data and was evaluated according to the Response Evaluation Criteria in Solid Tumors (RECIST, version 1.1). Chest CT and APCT were performed as follows: pre-dose on IP administration, pre-dose on day 1 of cycle 1, and at the completion of cycle 2. CT was performed as follows: pre-dose on IP administration, pre-dose on day 1 of cycle 1, and at the completion of cycles 2 and 4. Disease progression was established by chest CT, or APCT, performed within approximately four weeks before the initiation of study treatment. The efficacy parameters included complete response (CR), partial response (PR), stable disease (SD), progressive disease (PD), and not evaluable (NE), with frequencies and percentages reported. The objective response rate (ORR) is presented for each cohort and indicates the frequency, percentage, and 95% confidence interval of patients with the best overall response to CR or PR. Disease control rate (DCR) was provided for each cohort, and the frequency, percentage, and 95% confidence interval of patients with the best overall response of CR, PR, or SD were indicated. SD was confirmed through at least one observation after a minimum of six weeks from treatment initiation.

### Statistical analysis

All the statistical analyses were performed using SAS^®^ Version 9.4 (SAS Institute, Cary, NC, USA). Unless otherwise specified, all tests were conducted as two-sided tests at a significance level of 5%. The baseline and disease-related characteristics of patients, drug tolerability, laboratory test results, and vital signs are summarized by median for continuous data, and frequency and percentage for categorical data.

## Results

### Patients, dose escalation, and treatments

Seventeen patients were enrolled between October 2017 and April 2019. Three patients were excluded during the screening process. Therefore, 14 patients were included in this clinical trial. Fourteen patients who were administered the investigational drug at least once were included in the safety set. The characteristics of the participants in the safety set are shown in Table 2. Among the 14 patients, eight were male and six were female. The mean age ± standard deviation was 58.57 ± 8.28. Regarding ECOG performance status, eight patients (57%) had a score of 0, five (36%) had a score of 1, and one (7%) had a score of 2. In terms of primary tumor site, six patients (43%) had tumors in the rectum, six (43%) had tumors in the colon, one (7%) had tumors in the rectosigmoid, and one (7%) had tumors in the cecum. All patients had unresectable metastatic lesions: 13 had lung metastases, eight had liver metastases, eight had lymph node metastases, three had bone metastases, two had peritoneum/omentum metastases, and five had metastases at other sites. All patients experienced disease progression despite receiving second-line or higher systemic chemotherapy; six (43%) received second-line chemotherapy; five (36%) received third-line chemotherapy; and three (21%) received more than three lines of chemotherapy.

**Table 2:** Patient demographics and baseline characteristics

Characteristics	Patients (*n* = 14)
Gender, n (%)	
Male	8 (57)
Female	6 (43)
Age, years	
Median (range)	60 (44–73)
ECOG performance status, n (%)	
0	8 (57)
1	5 (36)
2	1 (7)
Metastatic sites at screening, n (%)	
1–2	7 (50)
≥ 3	7 (50)
Prior treatment, n (%)	
Surgery	14 (43)
Radiotherapy	5 (10)
Systemic therapy	
Oxaliplatin	13 (17)
Irinotecan	12 (17)
Bevacizumab	11 (20)
Cetuximab or panitumumab	5 (6)
Prior chemotherapy regimens, n (%)	
2	6 (43)
3	5 (36)
≥4	3 (21)

*ECOG*: Eastern Cooperative Oncology Group

Among the enrolled patients, 14 received at least one dose of OMT-110. The median relative dose intensity was 100%. The median treatment duration was 56 days (range 11–112 days): 73 days (11–112) for cohort 1, 56 days (56–56) for cohort 2, 37.5 days (14–112) for cohort 3, and 112 days (56–112) for cohort 4. Twelve patients were included in the DLT evaluation, excluding those who received less than 75% of the planned cycle 1 dose (21 doses) of OMT-110, excluding one from cohort 1 and one from cohort 3. Ten patients prematurely discontinued the treatment for the following reasons: disease progression or decision by the PI (*n* = 7), AEs (*n* = 2), and withdrawal of consent (*n* = 1).

### Safety

AEs were observed at each visit, and 54 TEAEs were reported. The most common TEAEs were nausea (n = 4, 29%), decreased appetite (n = 4, 29%), constipation (n = 3, 21%), asthenia (n = 3, 21%), dyspepsia (n = 2, 14%), pyrexia (n = 2, 14%), and hydronephrosis (n = 2, 14%) (Table 3). SAEs were pneumonia (n = 1, 7%), cardiac arrest (n = 1, 7%), and pyrexia (n = 1, 7%). These three events occurred in two individuals. One patient experienced pneumonia and cardiac arrest, leading to discontinuation and subsequent death 26 days after the first administration of OMT-110 due to PD. In another one patient, although the pyrexia was low-grade in severity, it was reported as an SAE because it resulted in hospitalization. A total of two patients experienced AEs that resulted in the permanent discontinuation of IP treatment. In one patient, weight loss (grade 1), asthenia (grade 2), and nausea (grade 2) were reported; although these events were not severe, the patient voluntarily withdrew consent. In the other patient, pneumonia (grade 3) was reported as a SAE and, as previously described, this patient was also discontinued IP administration. No adverse events caused death, serious adverse drug reactions (ADR), or ADR leading to permanent discontinuation of treatment. According to the investigator, among the 54 AEs, 52 were “definitely not related,” and two were “probably not related’; two were ADRs, nausea, and insomnia (grade 1).

**Table 3:** Treatment-emergent adverse events

Dose level	Cohort 1 (*n* = 4)		Cohort 2 (*n* = 3)		Cohort 3 (*n* = 4)		Cohort 4 (*n* = 3)		Total (*n* = 14)
	Grade 1–2	≥Grade 3	Grade 1–2	≥Grade 3	Grade 1–2	≥Grade 3	Grade 1–2	≥Grade 3	
Total	4 (26)	1 (2)	3 (13)	0	4 (13)	0	0	0	11 (54)
Gastrointestinal disorders	2 (4)	0	2 (5)	0	3 (3)	0	0	0	7 (12)
Nausea	2 (2)	0	1 (1)	0	1 (1)	0	0	0	4 (4)
Constipation	1 (1)	0	1 (1)	0	1 (1)	0	0	0	3 (3)
Dyspepsia	0	0	1 (1)	0	1 (1)	0	0	0	2 (2)
Abdominal pain	0	0	1 (1)	0	0	0	0	0	1 (1)
Ascites	0	0	1 (1)	0	0	0	0	0	1 (1)
Vomiting	1 (1)	0	0	0	0	0	0	0	1 (1)
General disorders and administration site conditions	3 (3)	0	0	0	2 (4)	0	0	0	5 (7)
Asthenia	2 (2)	0	0	0	1 (1)	0	0	0	3 (3)
Pyrexia	1 (1)	0	0	0	1 (2)	0	0	0	2 (3)
Chest discomfort	0	0	0	0	1 (1)	0	0	0	1 (1)
Musculoskeletal and connective tissue disorders	3 (3)	0	1 (2)	0	1 (1)	0	0	0	5 (6)
Flank pain	1 (1)	0	1 (1)	0	1 (1)	0	0	0	3 (3)
Back pain	1 (1)	0	0	0	0	0	0	0	1 (1)
Coccydynia	1 (1)	0	0	0	0	0	0	0	1 (1)
Myalgia	0	0	1 (1)	0	0	0	0	0	1 (1)
Metabolism and nutrition disorders	1 (1)	0	2 (2)	0	1 (1)	0	0	0	4 (4)
Decreased appetite	1 (1)	0	2 (2)	0	1 (1)	0	0	0	4 (4)
Renal and urinary disorders	1 (1)	0	1 (3)	0	1 (2)	0	0	0	3 (6)
Hydronephrosis	1 (1)	0	1 (3)	0	0	0	0	0	2 (4)
Calculus urinary	0	0	0	0	1 (1)	0	0	0	1 (1)
Hematuria	0	0	0	0	1 (1)	0	0	0	1 (1)
Infections and infestations	1 (1)	1 (1)	0	0	1 (1)	0	0	0	3 (3)
Bronchiolitis	1 (1)	0	0	0	0	0	0	0	1 (1)
Pneumonia	0	1 (1)	0	0	0	0	0	0	1 (1)
Tinea pedis	0	0	0	0	1 (1)	0	0	0	1 (1)
Vascular disorders	2 (4)	0	0	0	0	0	0	0	2 (4)
Hypertension	1 (3)	0	0	0	0	0	0	0	1 (3)
Hypotension	1 (1)	0	0	0	0	0	0	0	1 (1)
Investigations	1 (1)	0	1 (1)	0	0	0	0	0	2 (2)
Aspartate aminotransferase increased	0	0	1 (1)	0	0	0	0	0	1 (1)
Weight decreased	1 (1)	0	0	0	0	0	0	0	1 (1)
Nervous system disorders	1 (1)	0	0	0	1 (1)	0	0	0	2 (2)
Memory impairment	1 (1)	0	0	0	0	0	0	0	1 (1)
Neuropathy peripheral	0	0	0	0	1 (1)	0	0	0	1 (1)
Respiratory, thoracic, and mediastinal disorders	2 (2)	0	0	0	0	0	0	0	2 (2)
Cough	1 (1)	0	0	0	0	0	0	0	1 (1)
Dyspnea	1 (1)	0	0	0	0	0	0	0	1 (1)
Cardiac disorders	1 (1)	1 (1)	0	0	0	0	0	0	1 (2)
Cardiac arrest	0	1 (1)	0	0	0	0	0	0	1 (1)
Sinus tachycardia	1 (1)	0	0	0	0	0	0	0	1 (1)
Blood and lymphatic system disorders	1 (1)	0	0	0	0	0	0	0	1 (1)
Anemia	1 (1)	0	0	0	0	0	0	0	1 (1)
Injury, poisoning, and procedural complications	1 (1)	0	0	0	0	0	0	0	1 (1)
Radiation injury	1 (1)	0	0	0	0	0	0	0	1 (1)
Psychiatric disorders	1 (1)	0	0	0	0	0	0	0	1 (1)
Insomnia	1 (1)	0	0	0	0	0	0	0	1 (1)
Skin and subcutaneous tissue disorders	1 (1)	0	0	0	0	0	0	0	1 (1)
Dermatitis contact	1 (1)	0	0	0	0	0	0	0	1 (1)

Values are represented as the Number of Patients with adverse events (number of adverse events)

No DLTs were observed during clinical trials. However, the MTD of OMT-110 has not been determined yet. Therefore, a dose of 100 mg/day was selected for the phase 2 trial.

### Pharmacokinetics

**Fig. 1:**
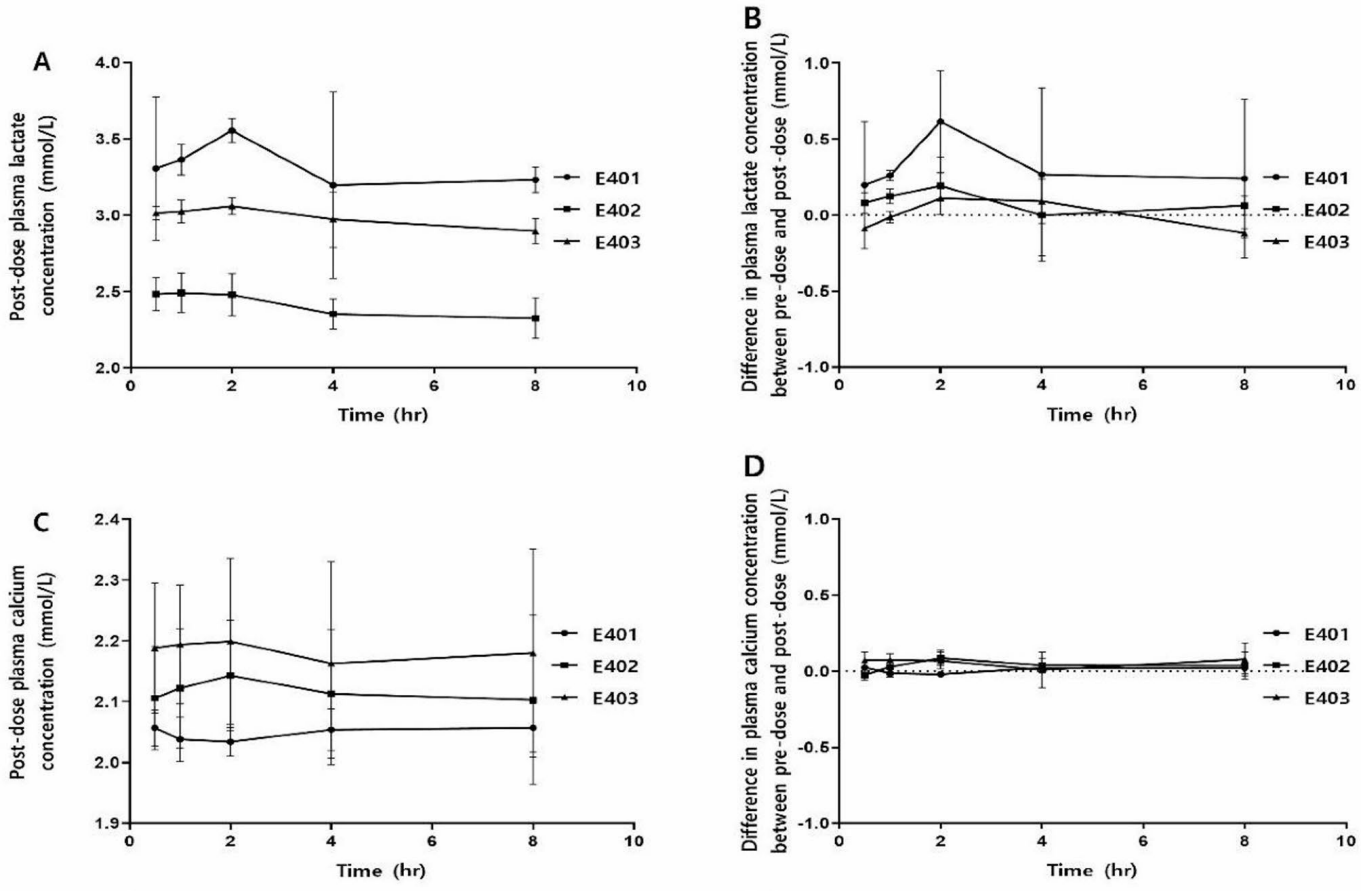
Pharmacokinetic evaluation of OMT-110: pre-dose sampling in cohort 4, cycle 1, days 1–2, and post-dose sampling in cohort 4, cycle 1, days 19–20. (**A**): Plasma concentration of lactate ions over time on day 20. (**B**): Difference in lactate concentration on day 20 compared to baseline. (**C**): Plasma concentration of calcium ions over time on day 20. (**D**): Difference in calcium concentration on day 20 compared to baseline

Pharmaceutical evaluations were conducted in three patients who received 100 mg of OMT-110, and the evaluation parameters included plasma concentrations of calcium and lactate ions. Peak plasma concentrations reached 2 h after administration, and baseline concentrations were restored by 4 h. No apparent accumulation of OMT-110 was observed from baseline until day 20. Pharmacokinetic assessments are summarized in Fig. 1.

### Pharmacodynamics

**Fig. 2:**
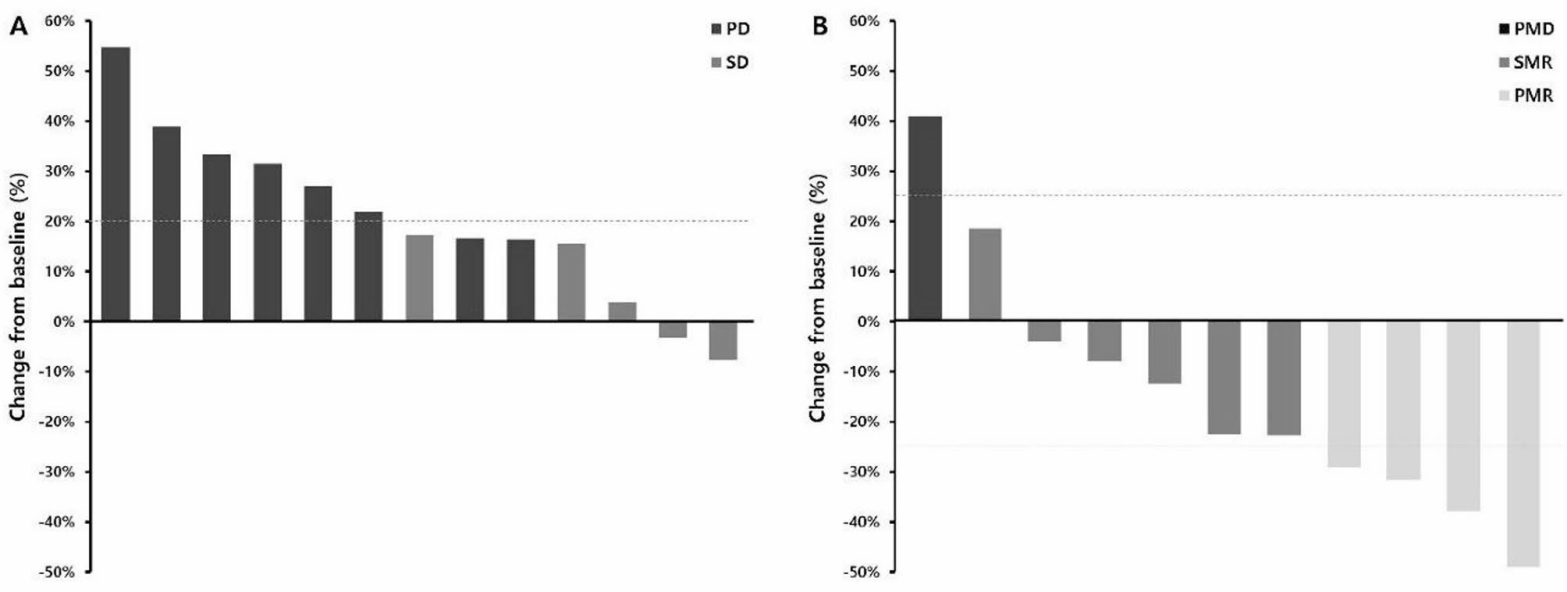
Difference from baseline (%) presented as ratios to baseline, listed based on best response criteria: (**A**) Subject data based on CT (*n* = 13) and (**B**) Subject data based on FDG-PET (*n* = 11). SUV Standardized uptake value

Among the enrolled patients, 11 underwent at least one follow-up FDG-PET/CT examination. The median duration of treatment was 56 days (range 45 to 112), and the median change in SUV_max_ for the best response was − 22.4% (-48.9–41.0%) (Fig. 2). Three to five lesions were observed in each patient, for a total of 52 lesions. The median change of individual lesions in SUV_max_ for the best response was − 20.8% (-100.0–99.4%). In one patient, no definite abnormal metabolism was observed in the two lesions measured using FDG-PET/CT. In terms of tumor responses according to EORTC criteria, the number of PMR, SMR, and PMD assessed for best response were 4, 6, and 1, respectively.

### Efficacy

Tumor response, according to RECIST, was evaluable in 13 of the 14 patients who underwent more than one follow-up CT scan. The median treatment duration (range) was 56 days (14–112). The best responses were SD in five patients and PD in eight patients. The overall DCR was 38%; two patients were evaluated based on non-target lesions because of the absence of target lesions. The treatment responses are summarized in Fig. 2.

Among the enrolled patients, nine (PPS) completed two cycles. The median treatment duration for PPS was 101 days (range: 56–112 days). The best responses were SD in five patients and PD in four patients. The median change in the best response rate was 16% (-8–27%). The DCR of the PPS was 56%. One of the nine patients was evaluated based on non-target lesions owing to the absence of target lesions. Among the enrolled patients, 13 (FAS) completed tumor response evaluations. The DCR of the FAS was 38%.

Among the enrolled patients, 11 had the target lesions. The median treatment duration was 56 days (range, 14–122 days), and the median change in the best response was 17% (-10–55%). A total of 29 target lesions were identified. The median change in the best response rate for these lesions was 16% (-20–56%). Among the target lesions, 13 were lung lesions with a median change in the best response of 14% (-20–56%), while 7 were liver lesions with a median change of 23% (-9–69%).

## Discussion

Based on the findings of the current study, no dose-limiting toxicities occurred, and a high compliance rate of 100% was observed for the relative dose intensity. Therefore, OMT-110 may be a safe drug that exceeds an MTD of 100 mg after subcutaneous injection. Consequently, a total dose of OMT-110 was recommended at 50 mg twice daily). The recommended phase 2 dose was determined based on tolerability and was supported by a pharmacokinetic assessment.

Of the 54 reported TEAEs, excluding two cases, all were mild with grade 1 or 2 severity. Among these, three serious TEAEs occurred in two patients: one from cohort 1 with pneumonia and cardiac arrest and another with pyrexia. The one patient in cohort 1 had a deterioration in their preexisting medical history (pneumonia) before the administration of OMT-110 in the clinical trial. The patient was hospitalized after 7 days of study enrolled due to preexisting pneumonia aggravation, and IP administration was discontinued after 11 days of treatment due to clinical deterioration. A pulmonology consultation determined that disease progression of pulmonary metastasis was the primary cause of the pneumonia aggravation. Throughout this period, tumor markers associated with colorectal cancer continued to rise, and imaging studies confirmed progression of metastatic colorectal cancer. Subsequent follow-up observations confirmed mortality owing to disease progression. This situation was exacerbated by the patient’s unfavorable preexisting health condition, which was characterized by consistent reporting of pain suspected to be related to cancer. The symptoms include abdominal pain, flank pain, and herpes zoster. Considering the overall clinical course, we determined that the two reported SAEs (pneumonia and cardiac arrest) in the single patient were attributable to the worsening of pre-existing conditions, namely metastatic colorectal cancer and pneumonia. Nonetheless, safety concerns should continue to be carefully monitored, and greater caution should be exercised in patient selection in future studies to avoid potential confounding factors.

One ADR occurred in one individual (7.14%), with two incidents. In Cohort 1, nausea and insomnia were observed once. These events were assessed as “probably not related” to the investigational drug and spontaneously resolved on the same day without specific treatment. The patient had preexisting health conditions, such as hyperlipidemia, radiation pneumonitis, and acute bronchitis. No serious ADR leading to the permanent discontinuation of OMT-110 or death was reported. The condition of the patient population was unfavorable, as the individuals were heavily pretreated for advanced mCRC. Even in the salvage treatment setting, grades three or higher SAEs were observed in two individuals (14.29%). However, the correlation of these TEAEs with the drug is considered “definitely not related” in 2 cases and “probably not related” in 1 case, indicating no significant association. Furthermore, no severe ADRs were observed, confirming the safety of the drug at the 0% level. In contrast, treatment with the conventional TAS-102 leads to grade 3 or higher neutropenia (38%), leukopenia (21%), and anemia (18%) [18–19]. In addition, according to the CORRECT trial, the most frequent regorafenib-related adverse events of grade 3 or higher were hand-foot skin reactions (17%), and almost all patients had various types and severities of treatment-related adverse events (93%) [20]. In the current study, OMT-110 demonstrated no severe toxicity; thus, this drug is safer than conventional agents.

Based on CT scans of 13 patients with FAS and nine patients with PPS, the DCRs were 38% and 56%, respectively, which underlines the effectiveness of OMT-110. The DCR was 44% with the conventional treatment, TAS-102 [18]. The ORR was 0%; however, many other studies have targeted mCRC and observed these low figures. In a study using TAS-102, the ORR was 1.6% [18]. Combination therapies such as regorafenib and nivolumab have been reported to have an ORR of 0% [21]. Considering the shift in the goals of anticancer therapy from cure to disease control and palliative care with mCRC progression, this result is not unfavorable [22]. Nonetheless, the observed DCR of 38% in the FSS and 56% in the PPS should be interpreted with caution, as it is based primarily on SD responses. Given the limited sample size and absence of ORR, these findings are considered exploratory and do not allow for definitive conclusions regarding the antitumor efficacy of OMT-110.

Using CT scans of patients with PPS, we observed a median increase in the size of the target tumors by 16%, suggesting potential disease control with OMT-110. Although the median change in tumor size was within the SD range according to the RECIST criteria, tumor growth was observed. However, an evaluation of the actual metabolic activity of cancer cells, conducted using FDG-PET/CT scans of nine patients from the PPS, revealed a median decrease in SUV_max_ of -13%. Considering the results of FDG-PET/CT scans and the proposed mechanism of action of OMT-110, this change could potentially reflect pseudo-progression, although this remains speculative. Pseudo-progression refers to tumor growth resulting from immune cell infiltration or inflammation induced by treatment, rather than true disease progression. In the case of OMT-110, the observed increase in tumor size may have been related to immunomodulatory effects. Importantly, this radiographic progression was not accompanied by clinical deterioration. However, we acknowledge that no immunologic biomarkers or pathological confirmation were obtained in this study, and therefore the hypothesis of pseudo-progression requires further validation in future studies incorporating immune profiling [23].

The plasma concentrations of calcium and lactate ions did not significantly differ between days 1 and 20 of treatment. This pharmacokinetic assessment suggested a lack of OMT-110 accumulation. Therefore, OMT-110 may reduce side effects, enhance safety, and exhibit efficacy in tumor therapy.

The pharmacodynamic assessment revealed an apparent reduction in cancer-specific metabolism. The median change in SUV_max_ of the tumors, measured via the FDG-PET/CT scan, was − 13% in the PPS. In both lesions, no definite abnormal metabolism was observed, indicating a decrease in glucose uptake and a return to normal levels. Considering the characteristics of cancer-specific metabolism with high glucose uptake [24], SUV_max_ may be an indicator for assessing cancer progression in patients with mCRC [25]. Furthermore, key factors significantly expressed in cancer-specific metabolisms, such as HIF-1 α, PKM2, and GLUT1, are positively correlated with high SUV_max_ expression. Based on these factors, the observed results imply an OMT-110-induced reduction in HIF-1 α and GLUT1 suppression. Within the PPS, one participant from cohort 4 had no FDG-PET/CT signals for two of the three lesions. The lack of a proportional correlation between dosage and efficacy is a limitation that must be assessed in a subsequent study.

The development of novel treatments for advanced mCRC is associated with several challenges. In this study, as mentioned earlier, the composition of the patient cohort posed difficulties compared to those of other studies. All participants had stage III-IV disease at the initial diagnosis and stage IV disease upon trial entry. Furthermore, one patient experienced early mortality due to comorbid conditions, irrespective of OMT-110, which was contrary to the expected lifespan during screening. Despite these limitations, this study demonstrated the efficacy and safety of OMT-110 based on the DCR and TEAE profiles.

OMT-110 appeared to be a safe treatment, as the MTD was not reached at effective doses. The observed discrepancies between the FDG-PET/CT and CT results, in relation to the context of its proposed mechanism of action, may suggest potential immunotherapy-related effects of OMT-110. However, further investigation is needed to explore and validate its possible immunomodulatory activity.

In conclusion, the cancer-specific metabolism inhibitor and potential immunotherapeutic agent OMT-110 exhibited remarkable safety and preliminary evidence of antitumor activity in a first-in-human dose-escalation study involving patients with advanced mCRC. OMT-110 showed a DCR of 56% within the PPS, suggesting that novel systemic therapeutic options are required for patients with advanced mCRC. Additional studies involving larger patient populations are necessary to evaluate the effective dose, therapeutic efficacy, safety, and potential immunomodulatory effects of OMT-110.

## Data Availability

The datasets used and/or analysed during the current study are available from the corresponding author on reasonable request.
